# Geosystemics View of Earthquakes

**DOI:** 10.3390/e21040412

**Published:** 2019-04-18

**Authors:** Angelo De Santis, Cristoforo Abbattista, Lucilla Alfonsi, Leonardo Amoruso, Saioa A. Campuzano, Marianna Carbone, Claudio Cesaroni, Gianfranco Cianchini, Giorgiana De Franceschi, Anna De Santis, Rita Di Giovambattista, Dedalo Marchetti, Luca Martino, Loredana Perrone, Alessandro Piscini, Mario Luigi Rainone, Maurizio Soldani, Luca Spogli, Francesca Santoro

**Affiliations:** 1 Istituto Nazionale di Geofisica e Vulcanologia – Sez. Roma 2, 00143 Rome, Italy; 2 INGEO Department, Università G. D’Annunzio, 66100 Chieti, Italy; 3 Planetek Italia srl, via Massaua 12, 70132 Bari, Italy; 4 SpacEarth Technology, 00143 Rome, Italy

**Keywords:** earthquakes, entropy, criticality, seismic precursors, Benioff strain, accelerated moment release

## Abstract

Earthquakes are the most energetic phenomena in the lithosphere: their study and comprehension are greatly worth doing because of the obvious importance for society. Geosystemics intends to study the Earth system as a whole, looking at the possible couplings among the different geo-layers, i.e., from the earth’s interior to the above atmosphere. It uses specific universal tools to integrate different methods that can be applied to multi-parameter data, often taken on different platforms (e.g., ground, marine or satellite observations). Its main objective is to understand the particular phenomenon of interest from a holistic point of view. Central is the use of entropy, together with other physical quantities that will be introduced case by case. In this paper, we will deal with earthquakes, as final part of a long-term chain of processes involving, not only the interaction between different components of the Earth’s interior but also the coupling of the solid earth with the above neutral or ionized atmosphere, and finally culminating with the main rupture along the fault of concern. Particular emphasis will be given to some Italian seismic sequences.

## 1. Introduction

Society advancement usually moves toward progress and modernization. However, the latter does not bring only positive things but they may also involve some vulnerability against natural hazards, much higher than in the past (e.g., [1]). Hurricanes, earthquakes (EQs), floods, tsunamis, and other kinds of catastrophes, are often out of human control and the consequences are unpredictable. They happen as extreme events on the planet causing destruction and deaths [2], and the occurrence of most of them looks as increasing dramatically in the last century [3]. Unfortunately, no strong remedy and rapid resilience are yet fully possible [4].

Among the possible solutions, one is to study and then understand how our planet works and what possible future sceneries are. To do this, we cannot limit our approach to a reductionist one, but we also need to study the Earth as a whole system, where all parts are nonlinearly interconnected and functional for the system to its evolution (e.g., [5]). The reductionist approach looks at Earth as a precise clock system where all components have their distinct own purpose (often called as the Laplacian point of view). With geosystemics [6,7], we can consider the planet as an ensemble of cross-interacting parts put together in order to reach the same ambitious goal that, at the present knowledge, seems to be rare in the Universe: to maintain life [8]. Earth system is both composed of living organisms and soft and hard engines, in a continuous balance and competition between life and death, heat and cold, complexity and simplicity, chaos and non-chaos. 

In this paper, we will remind the concepts of geosystemics and then apply them to EQs, through, among others, the Benioff strain, Entropy, temperature, etc., in the frame of a Lithosphere-Atmosphere-Ionosphere (LAI) coupling model, i.e., some quantities that are related to macroscopic features of the system under study. 

Although many efforts have been made towards a deeper knowledge of EQs, in terms of experimental, theoretical and numerical models (e.g., [9,10,11]), the evolution phases of an earthquake are not exhaustively explained yet. A possible explanation of this uncertainty is the lack of knowledge regarding the source initiation, the fracture mechanisms and dynamics of the crust (e.g., [12]). Moreover, each EQ initiates and develops in its proper geodynamical and lithological settings, thus giving an almost unique character to each event. Thus, to reach the knowledge necessary to recognize in advance the eventual rupture (failure) of the fault, which causes the occurrence of the EQ, is a greatly difficult task itself. Difficult as well, it is the possible explanation of the various and often weak phenomena affecting the above atmosphere and ionosphere, where even many external causes act to mix together signals, which are different in spectral content and amplitudes.

Despite all these difficulties, the eminent seismologist, [13], pointed out that some common physical mechanisms beneath the generation processes may act, although controlled by the local geodynamic forces and heterogeneities of the lithology [14]: this thought encourages the efforts towards a deeper knowledge of the physics behind such a complex phenomenon as the EQ.

If the process of rupture that causes the EQ is still plenty of open issues and unanswered questions (e.g., [15]), even more difficult is the understanding of the process of EQ preparation, although some efforts have been performed (e.g., [16,17,18]). It is thought that it may be accompanied by some exchanges of mass and energy, which can change the energy budget in the earth-atmosphere system over the seismogenic zone. In fact, scientific literature reports a wide variety of phenomena preceding EQs which have been studied extensively with the aim of finding some recurrent and recognizable patterns: induced electric and magnetic fields, groundwater level changes, gas and infrared (IR) electromagnetic emissions, local temperature changes, surface deformations, ionospheric instabilities (see [19,20] for more exhaustive reviews). 

With geosystemics introduced by the first author of this work, a great part of the paper is based on own contributions from mostly already published material. However, we attempted to give new insights on the idea of geosystemics, with also some unpublished own material or other researchers’ contributions. 

At first, we place the present view of geosystemics and show the application to some case studies. We then describe a possible physical model that attempts to explain the found results. We finally conclude with some feasible future directions and conclusions.

## 2. Geosystemics

Geosystemics looks at the Earth system in its whole focusing on self-regulation phenomena and interrelations among the parts composing our planet, possibly searching for the trends of change or persistence of the specific system or sub-system under study [6,7,21]. To this objective, geosystemics applies mainly the concepts of entropy and information content to the time series that characterize the phenomenon under study: as said by [22], to measure and understand the physical world, not only energy and matter are important, but also information. Interesting features of the complex system of interest to investigate are nonlinear coupling and new emergent behavior, self-regulation, and irreversibility as important constituents of the Earth planet. Entropy and information are very representative of the state and the possible evolution of the system under study [6].

No layer of the Earth system is really isolated, rather it interacts, in terms of transfer of energy or particles, with the other ones. This concept is more strengthened in the case of very powerful phenomena that release large energy in a short time, such as the earthquakes in the lithosphere (for an M7 earthquake, around 10^15^ Joule is released in some seconds), lightning strikes in the atmosphere (around 10^9^ J in microseconds), etcetera. For instance, the information exchanged between contiguous parts of the Earth system producing increased entropy would allow us to better recognize and understand those irreversible processes occurring in the Earth’s interior. As said by [21], “geosystemics has the objective to observe, study, represent and interpret those aspects of geophysics that determine the structural characteristics and dynamics of our planet and the complex interactions of the elements that compose it” by means of some entropic measures. 

Together with this, the approach will be based on multi-scale/parameter/platform observations in order to better scrutinize the particular sub-systems of Earth under study as much as possible. This is a fundamental issue of geosystemics because there is no better way to understand the behavior of a complex system than looking at it from as many perspectives and points of view as possible. Recent advanced examples to observe the planet are from satellites (e.g., [23]) and seafloors [24]. 

Geosystemics differs from the standard Earth System Science (e.g., [5,25,26,27]): for instance, in the way it is applied by means of entropic measures to different physical quantities, this because entropy is the only entity that can be used to have some clues on the next future. Please remind the second law of thermodynamics for which the entropy cannot decrease with time (e.g., [28]). This is related to the fact that a change of dynamical state requires some transfer of energy and some involved dispersal of it, mostly in terms of heat. The great advantage of this approach is that “the emergent dynamics may be extremely complex in detail, but the overall behavior of the system becomes simple as it is dominated by the overall constraints imposed by the thermodynamics of the system” [29] (p. 11).

In this paper, we will concentrate the attention to the application to EQ physics study and the possibility for intermediate and/or short-term prediction. Here, with the term “prediction”, we mean the possibility to make a prediction about EQ occurrence, magnitude and location, with small uncertainty, i.e., in a deterministic way, in contrast with the probabilistic approach used in EQ forecast (please see also in the next section for other details on this question). In particular, we will explore the present state-of-the-art of the seismological diagnostic tools based on a macroscopic point of view. As an EQ is the manifestation of a dramatic change of state of the lithosphere, geosystemics and entropy are powerful tools to study this kind of energetic phenomenon. Particular emphasis will be dedicated to the Shannon entropy [30]. Later on, we also see another one that quantifies the sense of the flow of information, the transfer entropy [31].

## 3. Main Seismological Diagnostic Tools

The Holy Grail in seismology is to reach the capability of giving a short-term prediction of large EQs thus eventually saving lives. Unfortunately, it is not an easy task as testified by the great all-out and full-scale effort made with this aim in many fields of research (even far from the traditional field of seismology) and the corresponding huge amount of scientific papers claiming or denying success or simply attempting some important steps forward towards the goal. However, despite many attempts, no significant success has been clearly counted [32].

Regarding the methods to make EQ “predictions”, we can classify them in (mainly) deterministic and (mainly) stochastic methods. The bracketed term “mainly” is placed because, actually, no method is only deterministic or stochastic. To be operative, we can define the latter methods as those that provide a forecast with some level of probability, for which the probability of no EQ is always different than zero, while the deterministic methods attempt to indicate the approaching of a large EQ with some level of confidence, i.e., with small uncertainty in space and time of occurrence, and magnitude.

Several statistical methods have been applied in the last decades to seismological data (mainly catalogs) with the aim of improving the knowledge on seismic phenomena. At present, the scientific community is involved in global projects to test and evaluate the performances of some well-established algorithms in different tectonic environments (see [33,34]). According to CSEP (collaboratory for the study of earthquake predictability), the most important steps of an earthquake prediction protocol are the following ones:

1. Present a physical model that can explain the proposed precursor anomaly.
2. Exactly define the anomaly and describe how it can be observed.
3. Explain how precursory information can be translated into a forecast and specify such a forecast in terms of probabilities for given space/time/magnitude windows.
4. Perform a test over some time that allows us to evaluate the proposed precursor and its forecasting power.
5. Report on successful prediction, missed earthquakes, and false predictions.

All these points are sequential, i.e., any mature precursor must sequentially satisfy them. However, if a precursor is at an initial stage of maturity, for instance, it has been just discovered in some case studies, it can satisfy only some of the first points, lacking the following ones. An early stage of the work on some novel precursors cannot exclude the publication of initial investigations. This is the case of most recent found precursors (e.g., entropy) that we will show below. 

In the present paper, we surely meet the first two points, leaving the other three points to other papers where a deeper and extended study is performed on a few but different precursors (e.g., [35,36]). We will mention something more about those works in a subsequent section.

In this part, we will focus our attention on the deterministic methods, which are essentially grounded on a systematic catalog-based recognition of some peculiar seismicity patterns in the given area of interest. A wide review of this topic is presented by [37]. In the following, we will describe *M8*, *RTP* (reverse tracing of precursors), *PI* (pattern informatics) and *R-AMR* (revised accelerating moment release). The latter method is the most recent and is the one we know much better because some of the present co-authors have introduced the corresponding technique [38]. For this reason, we will dedicate a specific section to it.

### 3.1. M8

*M*8 owes its name to the fact that it was designed as a retroactive analysis of the seismicity preceding the greatest (M8+) EQs worldwide (e.g., [39,40]). Some spatio-temporal functions are introduced in order to describe the seismic flow in a target area (wider than the earthquake source). The M8 takes into account only mainshocks, which are described by a 6-component vector, i.e., time (t), latitude, longitude, depth(h), magnitude (M), and the function B(*e*) that corresponds to the number of aftershocks that occurred after the first *e* days after the mainshock. The function N(t) is the intensity of the earthquake flow and it represents the current state of seismic activity. L(t) is the deviation of N(t) from the long term trend. As the earthquake occurrence rate depends on the zone, the method normalizes the magnitude of the event and the earthquake flow becomes constant, usually 10/year and 20/year, so it takes into account 6 years of the time interval. The algorithm then recognizes a well-established criterion, defined by extreme values of the phase space coordinates, as a vicinity of the system singularity. When a trajectory enters the criterion, the probability of an extreme event increases to the level sufficient for its effective provision, so an alarm or a TIP, “time of increased probability”, is declared. This algorithm can be modified for lower magnitudes and particular regions (e.g., CN8).

### 3.2. The Reverse Tracing of Precursors (RTP)

The RTP is a method for medium-term (some months in advance) EQ prediction [41], which is based on a hierarchical ensemble of premonitory seismicity patterns. These patterns are: (1) “precursory chains” that are related to the correlation length (e.g., [42,43]), (2) “intermediate-term patterns” that could be related to some accelerating seismicity (e.g., [44]) and (3) “pattern recognition of infrequent events” that take into account several “opinions” to decide the validity of the calculated chain of events. If a sufficient number of “votes” is accumulated, then the chain is considered precursory [41]. Some past EQs seem to have been predicted 6 to 7 months in advance, although few false alarms also happened. Critical aspects are related to the predicted “area of alarm” that seems very large for a realistic application. RTP has already evaluated by the gambling score, showing apparently only marginal or no significance in predicting earthquakes [45]. However, [46] criticized this conclusion, affirming that: “The statistical analysis of any prediction method with few target events and a short monitoring period is premature (this is the case of RTP)”. 

### 3.3. Pattern Informatics (PI)

The *PI* is a technique for quantifying the spatio-temporal seismicity rate changes in past seismicity (e.g., [47,48]). In [49] the authors derive a relationship between the “PI index” and stress change (e.g., [50]), based upon the crack propagation theory. In practice, the PI method measures the change in seismicity rate at each box of a pre-defined grid, relative to the background seismicity rate, through the division of the average rate by the spatial variance over all boxes. Then it identifies the characteristic patterns associated with the shifting of small EQs from one location to another through time prior to the occurrence of large EQs [49]. Results are given in terms of mapping the “PI anomalies” which are located where a new large EQ can be expected. [51] proposed a modification of PI by using complex eigenfactors, explaining the EQ stress field as obeying a wave-like equation. 

## 4. Shannon Entropy and Shannon Information

The Shannon Entropy *h*(t) [30] is an important tool for the space-time characterization of a dynamical system. In general, for a system characterized by *K* possible independent states, this entropy is defined in a certain time *t* as follows:

(1) $$h\left(t\right)=-\overset{K}{\underset{i=1}{\sum }}p_{i}\left(t\right)\cdot \log p_{i}\left(t\right)$$

where $p_{i}\left(t\right)$ is the probability of the system to be at the *i*-th state. For convenience, we impose $\underset{i}{\sum }p_{i}=1$ and $\log p_{i}=0$ if $p_{i}=0$ to remove the corresponding singularity. Although the base of the logarithm could be any, we will use later on the decimal one for the logarithm.

In literature, we can find a large number of physical interpretations of the Shannon entropy. We consider here what we think it is the simplest one: it is a non-negative measure of our ignorance about the state of the system of concern. The Shannon entropy has great importance in evaluating and interpreting the behavior of complex systems like the Earth, in general, and EQs, in particular. On the other hand, we find in literature also the Shannon Information, *I*(*t*), which is simply related to *h*(*t*) by the simple relation *I*(*t*) = −*h*(*t*). Consequently, the Shannon information is a negative quantity that measures our knowledge on the state of the system when we know only the distribution of probability *p*(*t*) [52]. Thus, this quantity measures our decreasing ability to predict the future evolution of the system under study. 

## 5. Gutenberg-Richter Law and *b*-Value 

The Gutenberg-Richter (GR) law has a central role in seismology [53]. It expresses the logarithm of the cumulative number *n* of EQs with magnitude *m* equal to or larger than a magnitude *M*:

(2) $$\log_{10}n\left(m\geq M\right)=a-b\cdot M$$

as a simple linear function of the magnitude *M*; *a* and *b* are two constant parameters for a certain region and time interval, characterizing the associated seismicity; in particular, *b* is the negative slope of the above cumulative distribution and typically *b* ≈ 1. Very soon it was recognized the importance of estimating the *b*-value as an indicator of the level of stress in a rock from laboratory experiments [54], and only later the relationship was confirmed for EQs [55]. 

Ref. [56] was the first to provide a simple expression to estimate *b* by means of the maximum likelihood criterion (with a correction proposed by [57]:

(3) $$b=\frac{\log e}{\bar{M}-M_{min}+\cdot /2}$$

whose uncertainty is: $\pm b/\sqrt{N}.$

*N* is the total number of analyzed EQs; *e* = 2,71828 … is the Euler number, while $\bar{M}$ is the mean value of the magnitudes of all considered EQs; *M*_min_ is the minimum magnitude used in the *b*-value evaluation; Δ is the resolution involved in the magnitude estimation, normally Δ = 0.1. Usually, the *M_min_* is the magnitude of completeness of a seismic catalog, i.e., the magnitude threshold at which or above the corresponding seismic catalog includes all occurred EQs in the region.

## 6. Entropy and EQs

Now we apply the concept of Shannon entropy to EQs. Most of this section is based on [58], with some extension in order to clarify some concepts.

Given a sequence of EQs (in the form of a seismic catalog or a seismic sequence within a certain region) with non-negative (and normalized) probability *P_i_* to have activated a certain *i*-th class of seismicity characterized by some range of magnitudes, the associated non-negative Shannon entropy *h* can be defined as [58]:

(4a) $$h=-\sum_{i=1}^{K}P_{i}\cdot \log P_{i}\geq 0.$$

Since Equation (4a) is applied to a discrete number of states, *h* is also called *discrete Shannon entropy*. It can be considered a reliable measure of uncertainty and missing information about the system under study.

Actually, the values of magnitude can assume a continuous range (in theory from *M_min_* to infinity) then the discrete definition (4a) becomes an integral definition [30,59]:

(4b) $$H=-\int_{M_{min}}^{\infty }p\left(M\right)\cdot \log p\left(M\right)dM$$

where *H* is now called *continuous* (or *differential*) *Shannon entropy* to be distinguished from *h*, and *p*(*M*) is the probability density function (*pdf*) of the magnitudes *M*, such as $p\left(M\right)=\frac{d}{dm}\sum_{i,m\leq M}P_{i}\left(m\right)$ and $\int_{M_{min}}^{\infty }p\left(M\right)dM=1$.

It is worth noticing that moving from the discrete definition (4a) to the continuous (4b), *H* loses the property of non-negative owned by *h*; thus *H* can assume also negative values (e.g., [59]). Since this is not evident from the work by [58], we will spend here some words about it.

The two definitions of the Shannon entropy are related by the following equation:

(5) H=h+logδ

where δ is the sampling step of the continuous *pdf* in order to let it discrete in *h* (e.g., [60]). It is evident from (5) that when δ tends to zero, *H* will diverge to −∞. Thus, the continuous entropy *H* is not a limit for δ → 0 of the discrete Shannon entropy *h* and, consequently, it is not a measure of uncertainty and information. Nonetheless, the continuous Shannon entropy can be used to measure differences in information [59].

However, when the classes of magnitude are loose, e.g., for δ ≈ 1 we will have *H* ≈ *h*: [58] considered δ = 0.5 so the difference between discrete and continuous entropies was only 0.3. 

Ref. [58] have shown that if the *p*(*M*) is the GR probability distribution, then *H* can be expressed in terms of the *b*-value:

(6) H≈0.072−logb

The derivation of (6) follows from the probability density function corresponding to the Gutenberg-Richter law

(7) $$p\left(M\right)=\frac{b\cdot 10^{-b\left(M-M_{min}\right)}}{\log e}\;\mathrm{with}\;M\geq M_{min}.$$

Hence, by imposing $M_{min}=M_{c},$ we get

(8) $$\begin{array}{l} H=-\overset{\infty }{\underset{M_{c}}{\int }}p\left(M\right)\cdot \log p\left(M\right)dM=-\overset{\infty }{\underset{M_{c}}{\int }}\frac{b\cdot 10^{-b\left(M-M_{c}\right)}}{\log e}\cdot \log\left(\frac{b\cdot 10^{-b\left(M-M_{c}\right)}}{\log e}\right)dM=\\ =-\frac{b}{\log e}\overset{\infty }{\underset{M_{c}}{\int }}10^{-b\left(M-M_{c}\right)}\left[\log b-b\left(M-M_{c}\right)-\log\left(\log e\right)\right]dM=\\ =-\frac{b}{\log e}\left\{\left[\log b-\log\left(\log e\right)\right]\overset{\infty }{\underset{M_{c}}{\int }}10^{-b\left(M-M_{c}\right)}dM-b\overset{\infty }{\underset{M_{c}}{\int }}\left(M-M_{c}\right)10^{-b\left(M-M_{c}\right)}dM\right\}=\\ =-\frac{b}{\log e}\left\{\left[\log b-\log\left(\log e\right)\right]\frac{\log e}{b}-b\frac{{\left(\log e\right)}^{2}}{b^{2}}\right\}=-\log b+\log\left(\log e\right)+\log e, \end{array}$$

i.e., Equation (6). It provides an alternative explanation of the typical decrease of *b*-value as seismic precursor (e.g., [61,62], for the case of 6 April 2009 M6.3 L’Aquila earthquake in Central Italy) as an increase of entropy culminating almost at the mainshock [58].

## 7. Entropy and Critical Point Theory

An ergodic dissipative system can have a critical point where the system undergoes through a transition. The ergodic property means that the system averages in real 3D space are equivalent to averages in the ideal reconstructed phase space (e.g., [63,64]). As an example, we can remind the behavior of the specific heat around a critical point occurring at temperature T_λ_ degree, when the system approaches the critical temperature as a power law. In addition, if the system changes its temperature linearly in time, the same plot is expected versus time [65]. 

**Figure 1:**
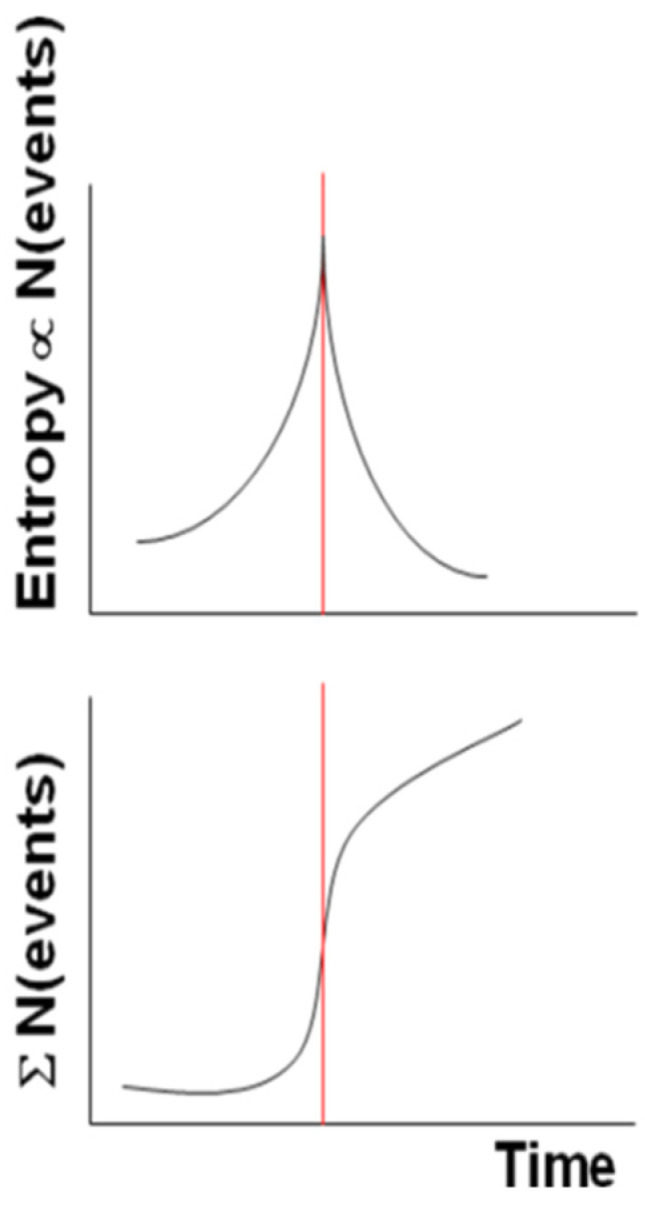
Idealized Shannon entropy (above diagram) and a cumulative number of events (bottom diagram) for a dissipative system around its critical point, indicated by the vertical red line.

More generally, if we replace the increasing temperature with the system entropy, then the system reaches its critical point (vertical red line in Figure 1) at the largest entropy and approaches it with an accelerating power law in its cumulative of punctuated events (we intend here for an “event” as an anomalous behavior of the system evolution, e.g., when its signal level is larger than a certain number of standard deviation, σ, e.g., 2.5 σ). 

After the critical point, the curve behaves as a decelerating power law. Figure 1 depicts both the idealized behaviors for the entropy and the cumulative number of events.

We will see in the following how these patterns are reproduced in the case studies of some Italian seismic sequences.

In seismology, the occurrence of an EQ can be considered as a phase transition, for example in the natural time domain the variance k_1_ is taken as an order parameter (e.g., [66]). As we defined (and applied) the Shannon entropy, we will show that it is a reliable parameter to characterize the critical point in both the two Italian case studies. It can be considered a parameter similar to other order parameters, with the difference the latter are usually approaching a minimum value while the Shannon entropy gets the largest one.

## 8. Entropy Studies of Two Italian Seismic Sequences

In this part, we show two case studies in Italy: the 2009 L’Aquila and the 2012 Emilia seismic sequences, both producing a main-shock of around M6 (precisely local and moment magnitudes, ML5.9 and Mw6.2 for L’Aquila and local magnitude ML5.9 for Emilia). Main characteristics of the two seismic sequences are given in Table 1. The first case was already analyzed and discussed by [58]. However, we will make here some alternative/complementary analyses, with respect to those already published. The second case study is original and never published so far.

In both cases we considered all earthquakes with a minimum magnitude equal to (for entropy analysis) or well above (for R-AMR; see Section 9) the completeness magnitude of the earthquake catalogs, that was found of M1.4+ and M2+ for L’Aquila and Emilia earthquake sequences, respectively. 

### 8.1. The 2009 L’Aquila Seismic Sequence

**Figure 2:**
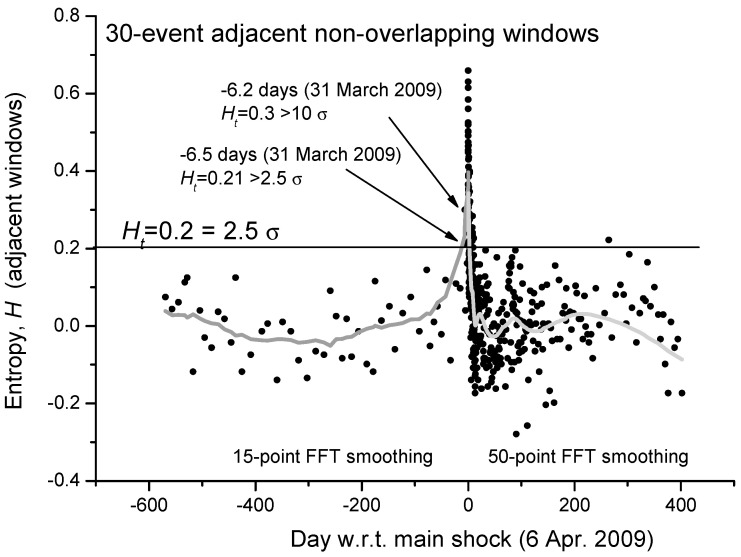
Shannon entropy for L’Aquila seismic sequence from around 1.5 years before the main-shock to around 1 year after, calculated for a circular area of 80 km around the main-shock epicenter. Each point is the entropy analysis based on non-overlapping windows, each composed by 30 foreshocks. The gray curve defines a reasonable smoothing of the entropy values: 15-point FFT before the main-shock and 50-point FFT smoothing after the main-shock. The different kind of smoothing is related to the different rate of seismicity before and after the main-shock. It is interesting how the smoothed curve reproduces the expected behavior of a critical system around its critical point. (Adapted from [67]).

As mentioned in [58], Shannon entropy can be estimated in three different ways: cumulative, moving overlapping or distinctively temporal windows. For the first case study, i.e., the 2009 L’Aquila (Central Italy) seismic sequence, we will consider adjacent non-overlapping moving windows. In Figure 2 we show the estimation of the Shannon entropy based on non-overlapping windows of 30 M1.4+ seismic events occurred in a circular area of 80 km around the main-shock epicenter. The low number of events used for the analysis in each window was chosen to better follow even shorter fluctuations of entropy, especially for the foreshocks. It is interesting that two distinct entropy values before the main-shock occurrence are larger than the threshold H_t_ = 2.5 σ (the mean value of entropy, <*H*>, is practically zero). To better visualize the mean behavior of entropy, the gray curve defines a reasonable smoothing of the entropy values: 15-point FFT before the main-shock and 50-point FFT smoothing after the main-shock. The different kind of smoothing is related to the different rate of seismicity before and after the main-shock. It is interesting to notice that the smoothed gray curve of the Shannon entropy reproduces the expected behavior of a critical system around its critical point, with the main-shock as a critical point.

**Figure 3:**
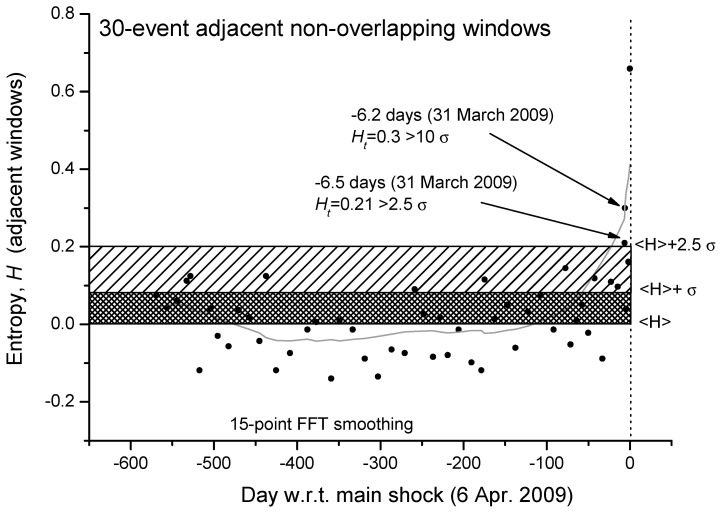
Details of the Shannon entropy for L’Aquila seismic sequence from around 1.5 years before the main-shock to the main-shock occurrence. Each point is the entropy analysis based on non-overlapping windows, each composed by 30 foreshocks. The mean value of the entropy, <H>, which is almost zero, and one and two standard deviations are also shown. The gray curve defines a reasonable smoothing of the entropy values with 15-point FFT.

We can even analyze in more detail the same curve but expanded in the period before the main-shock (Figure 3). We confirm that, around 6 days before the main-shock, there is the persistence of two consecutive values of entropy greater than 2.5σ (the larger value is even greater than 10σ). An interesting question to better investigate in more case studies will be: could this persistence of larger values of entropy be considered a reliable precursor of the imminent main-shock?

### 8.2. The 2012 Emilia Seismic Sequence

In this specific case study, we will consider moving and partially overlapping windows, each composed of around 200 seismic events and overlapping of 20 events. This kind of analysis allows us to have directly a smoother curve of entropy, without resorting to a subsequent smoothing operation as done instead in the previous case.

**Figure 4:**
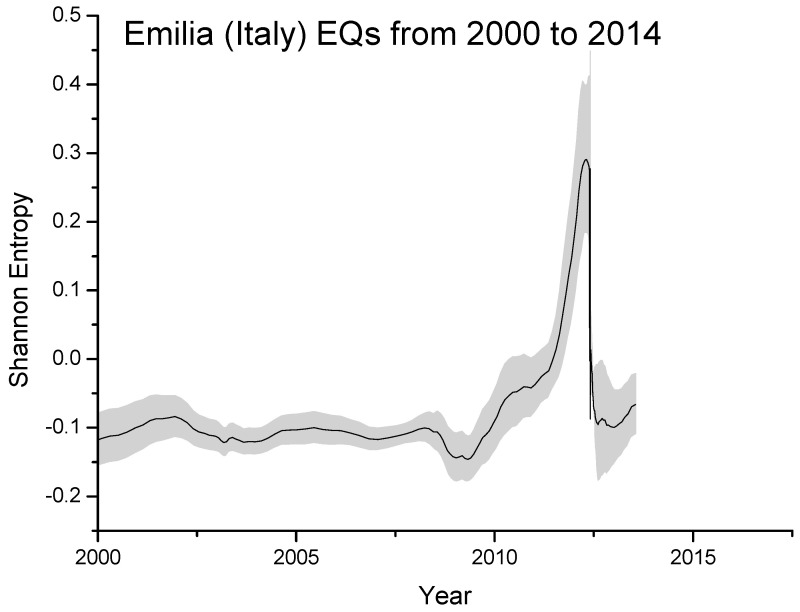
Shannon entropy for the Emilia seismic sequence from 2000 to 2014. The significant increase from around 2010, with the maximum at around the main-shock occurrence, is expected to be real. The gray area defines the statistically estimated (one standard deviation) error in computing the entropy.

In Figure 4 we plot the Shannon entropy for the Emilia seismic sequence from 2000 to 2014, as estimated overall M2+ EQs occurred around 150 km from the first major EQ. The significant increase starting around 2010 is probably real and related to the preparation phase of the two major EQs occurred on 20 and 29 May, 2012 with local magnitudes 5.9 and 5.8, respectively, where the entropy reaches the maximum value (in this case around 0.3). The gray area defines the estimated error in computing the entropy.

As a general remark of this section, it is true that we have applied the entropy analysis to two case studies only, but in most occasions, we could extend the found results by analyzing *b*-value to the entropy, via Equation (6). The introduction of the Shannon entropy in the analysis of a seismic sequence provides a more physical and statistical meaning to the potential precursory decrease of the *b*-value in terms of an increasing entropy of the underlying physical system.

Precursory entropy changes have been also observed when analyzing the seismic data in natural time [66] and using for the computation a sliding window comprising a number of events that occur within a few months or so, which is the lead time of the precursory seismic electric signal activities [68] detected before major earthquakes. For example, almost three months before the 2011 M9 Tohoku earthquake in Japan it was recently found [69] that the entropy change under time reversal exhibited an unprecedented minimum. Such a minimum has been also observed before the M8.2 Mexico earthquake that occurred on 7 September 2017 [70].

## 9. Accelerated Moment Release Revisited: The Case of L’Aquila and Emilia EQs

Reference [71] proposed a simple way to estimate the strain-rebound increment, ε_i:_

(9) $$s_{i}=\sqrt{E_{i}}=k_{i}\cdot \varepsilon_{i}$$

where *E_i_* is the energy released by the EQ, i.e., 10^αM + β^ (α = 1.5, β = 4.8 for energy expressed in Joule, although Benioff used slightly different values), and *k_i_* = (μ*PV_i_*/2)^0.5^ (μ = shear or rigidity modulus, *V_i_* = volume of the i-th fault rocks, *P* is the fraction of energy transmitted in terms of seismic waves; usually it is considered *P* ≈ 1). This theory is based on [72] arguments of the elastic rebound. 

To take account the cumulative effect of a series of *N* EQs at the time *t* of the last *N*-th EQ, Benioff introduced therefore what is now called the cumulative Benioff strain:

(10) $$s(t)=\sum_{i=1}^{N(t)}s_{i}=\sum_{i=1}^{N(t)}\sqrt{E_{i}}=10^{\beta^{'}}\sum_{i=1}^{N(t)}10^{0.75M_{i}}$$

with β’ = β/2 = 2.4. It is important to notice that, according to [71], the cumulative strain (8) is that accumulated on the fault under study.

Extending the meaning of (10) to the strain accumulated over a larger area around the epicenter, [73] obtained interesting results with the so-called accelerating moment release (*AMR*) approach that consists in fitting the cumulative value *s*(*t*) expressed as in Equation (8), with a power law in the time to failure *t_f_*, i.e., the theoretic time of occurrence of the main shock: *s*(*t*) = *A* + *B*(*t_f_* − *t*)*^m^*, where *A*, *B* and *m* are appropriate empirical constants (*m* is expected between 0 and 1: typical value is 0.3; [37]). The fitting process gives as an outcome the time *t_f_* together with the expected magnitude, which is related to either *A* or *B*:

(11a) $$M_{p}(A)=\frac{\log(\Delta s_{last})-\beta '}{0.75}$$

where $\Delta s_{last}=A-s_{last}$ and *s_last_* are the cumulative Benioff strain at the last precursory event considered (namely the *N*-th EQ). In this expression, one speculates that the main-shock will be the next EQ striking after the *N*-th, but the occurrence of many smaller EQs after the last analyzed shock and before the predicted time *t_f_* cannot be excluded. 

An alternative formulation, based on the parameter *B,* has been given by [74]:

(11b) $$M_{p}(B)=\frac{\log\left|B\right|-\beta '-0.14}{0.738}.$$

Criticism to this method came from [75] who pointed out the arbitrariness in the critical choice of the temporal and spatial criteria for data selection, i.e., the initial precursory event of the *AMR* curve and the extension of the inspected region. On the other hand, [76] explained the AMR phenomenon under the view of complexity principles.

To circumvent this criticism, [38] introduced what they called R-AMR, i.e., the revised accelerating moment release (R-AMR), as a better way of applying the AMR by weighting the EQs magnitudes in a certain area, according to an appropriate attenuation function *G* = *G*(*R*), where *R* is the distance of a given EQ epicenter with respect to the impending slipping fault. In particular, the Benioff Strain produced at the fault level is expressed by a reduced Benioff strain *ŝ*(*t*) = *s∙G* called “reduced” because the action of the function *G*, which is normally less than unity (i.e., *G* ≤ 1), is to diminish the value of the typical Benioff strain, normally according to the distance *R* from the center of the region of study. As an area of interest, a circle is taken with the corresponding Dobrovolsky radius, r(km) = 10^0.43M^ with M = EQ magnitude [16].

Thus, the expression for the cumulative reduced strain becomes:

(12) $$\tilde{s}(t_{})=\sum_{i=1}^{N(t)}{\tilde{s}}_{i}=\sum_{i=1}^{N(t_{i})}\sqrt{E_{i}}\cdot G(R_{i})=10^{\beta '}\sum_{i=1}^{N(t_{i})}10^{0.75M_{i}}G(R_{i}).$$

Ref. [38] applied with success their revisited method to the three most important seismic sequences occurred in Italy in the previous ten years with respect that publication. In addition, they also showed that, for a particular seismic swarm (i.e., with no mainshock), R-AMR performs better than AMR, not providing a false alarm.

### R-AMR for the 2009 L’Aquila and 2012 Emilia Seismic Sequences

**Figure 5:**
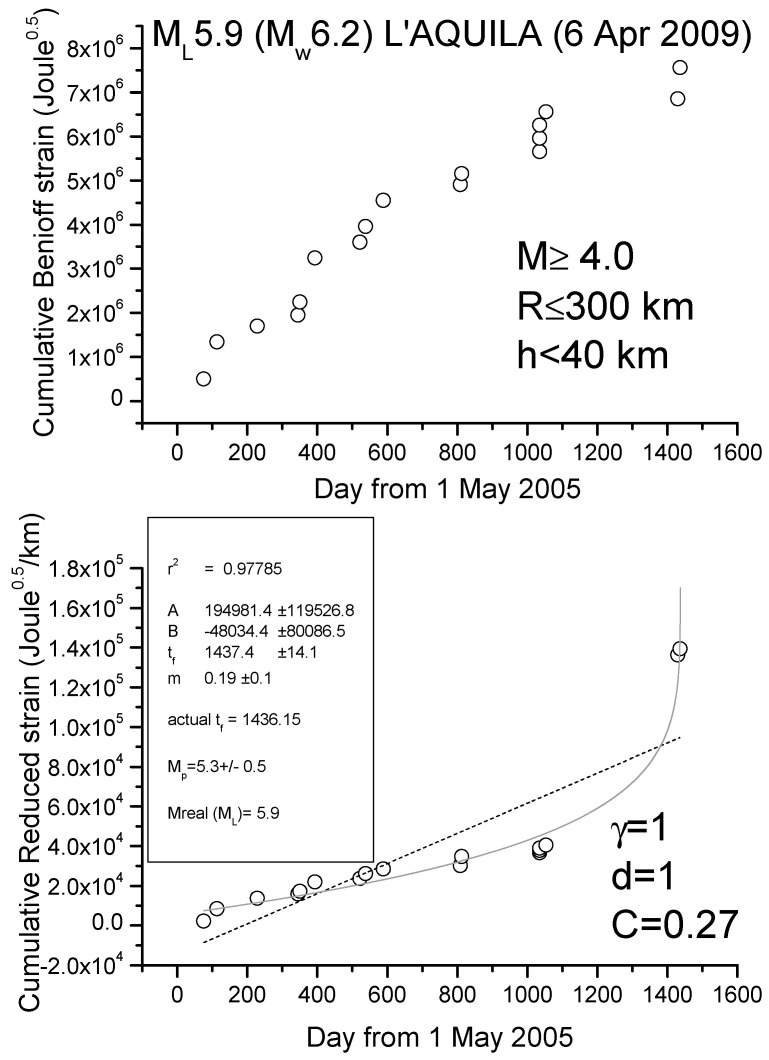
Analyses of L’Aquila seismic sequence *M* ≥ 4 EQs (main-shock not shown and not used in the analysis): (top) ordinary AMR method; (bottom) R-AMR method. The dashed line represents the best linear fit, while solid gray curve is the best power law fit. Results of the fit are shown in the frame inside the graph at the bottom; r^2^ is the coefficient of determination, providing a measure of the quality of the fit (the closer to 1, the better the fit).

**Figure 6:**
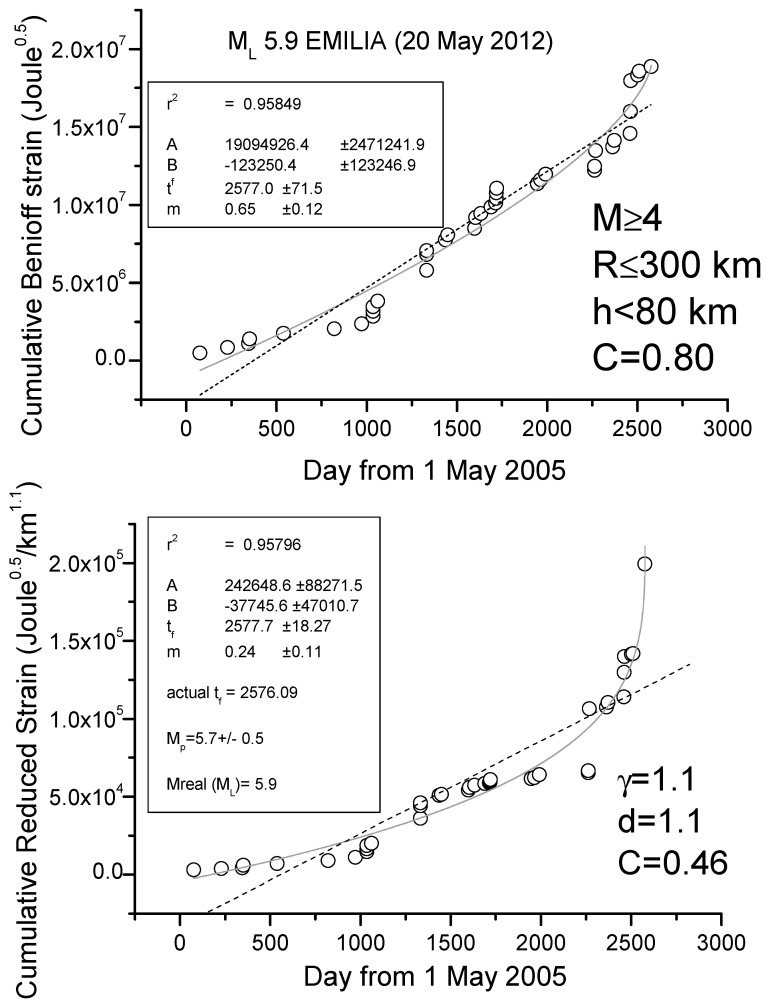
Analyses of Emilia seismic sequence *M* ≥ 4 EQs (main-shock not shown and not used in the analysis): (top) ordinary AMR method; (bottom) R-AMR method. Here the ordinary AMR also showed a little acceleration (*C*-factor = 0.80) but the R-AMR version is much better (*C*-factor = 0.46). The dashed line represents the best linear fit, while the solid gray curve is the best power law fit. Results of the fit are shown in the frames inside the graphs; r^2^ is the coefficient of determination, providing a measure of the quality of the fit (the closer to 1, the better the fit).

We show here two case studies, L’Aquila and Emilia seismic sequences, where the application of R-AMR is made much simpler than the one firstly proposed in [38]. Although that way of applying of R-AMR is more rigorous because all EQs above the minimum magnitude of completeness are considered, we show here that a simpler application is also possible, where considering a very simple attenuation function of the form G(R_i_) = d/R_i_^γ^, with d (normally 1km), R_i_ in km and with γ ≈ 1, at the cost of considering a larger minimum magnitude threshold of around M4. Figure 5 and Figure 6 show the results for the cases of L’Aquila and Emilia sequences, respectively, where we apply to all shallow (depth h ≤ 40 and h ≤ 80 km, respectively) M4+ EQs both AMR and R-AMR analyses (top and bottom of each figure, respectively). Then, we consider a 300 km size for the regions where we applied R-AMR analysis. This size is comparable with the corresponding Dobrovolsky’s radius. Both the analyses stop well before the main-shocks that are not considered in the calculations. We notice that the time of preparation is rather long for both sequences, i.e., practically starting at the beginning of the whole period of investigation (May 2005). This fact could be simply interpreted as the larger foreshocks anticipate the beginning of the seismic acceleration with respect to the smaller ones, which were the most in the previous analyses in [38]. 

The goodness of the power law fit with respect to the linear regression can be quantified by the C-factor which is the square root of the ratio between the RMS of the power law and the RMS of the best linear fit [77]: the lower the C-factor than 1, the better the power law fit is with respect to the line.

We find a clear seismic acceleration for both seismic sequences, quantified by a low value of C (0.27 for L’Aquila sequence and 0.46 for Emilia sequence) and a great determination coefficient (r^2^ > 0.95 in both cases). In addition, the predicted magnitudes are comparable with (although lower than) the real ones. In both cases, the beginning of clear acceleration starts around 1.5 years before the main-shock.

The above cases represent two of the four seismic sequences happened in Italy in the last 15 years. Another seismic sequence, occurred in south Italy in 2010 and culminated with an M5 in the Pollino area, shows an analogous acceleration before the mainshock [38]. Only the most recent seismic sequence of the Amatrice-Norcia (Central Italy) earthquakes in 2017 had neither acceleration nor foreshocks before the first major earthquake (24 August 2016 M6 Amatrice earthquake). Therefore, the two case studies shown here are very representative of the most recent seismicity in Italy that has expressed in terms of a series of earthquakes culminating with a mainshock.

## 10. Lithosphere-Atmosphere-Ionosphere Coupling (LAIC)

Geosystemics [6,7] sees the planet in its entireness, where all geo-layers “communicate” each other, in terms of exchange of matter and/or energy, i.e., what [22] called with the more generic term of “information”. In the last two decades, an important model, so-called lithosphere-atmosphere-ionosphere coupling (LAIC) proposes that some precursory anomalies can appear in the atmosphere and/or ionosphere before a large EQ, during its preparation phase (e.g., [78]). 

The state of the ionosphere is particularly sensitive to the LAIC. Its presence as an ionized layer at 50–1000 km altitude above the Earth’s surface is important to detect any electromagnetic change in the circumterrestrial environment [79]. Comprehensive reviews of the papers that describe the measurements of the seismo-ionospheric signals are reported in [20] and [80]. In addition, [81] made a discussion on the temporal and spatial variability of the ionospheric precursor summarizing the results obtained by a large number of authors so far. In particular, they describe in detail what is the role of the global electric circuit in transferring information from the Earth’s surface up to the ionosphere. 

The finding of atmospheric anomalies prior to large EQs is more recent and widely debated as well.

In this section, we remind some of those phenomena, the nature and characteristics of which are more directly of interest for the understanding of LAIC.

### 10.1. Pre-EQ Ionospheric Evidences from Ground-Based Observations

A coupling (post-seismic) effect of an EQ to the above atmosphere is already well known: it can appear just after the occurrence of a sufficiently large event, and it is related to the possibility of observing the effect of the propagation of acoustic-gravity waves in the ionosphere (e.g., [82]). Ref. [83] reported one of the first reports of total electron content (TEC) anomalies due to coseismic gravity waves. They found an anomaly in vTEC that propagated from near field to around 1000 km just after the M7.9 12 May 2008 Wenchuan earthquake. Recently, this effect has been clearly detected as wave-like fluctuations of the TEC in ionosphere 21 min after the April 25, 2015, M7.8 Nepal EQ ([84]; last access on 17 November 2018).

Important precursory effects of LAIC before large EQs can be detected in the ionosphere from ground-based observational systems like ionosondes and GPS (global positioning system)/GNSS (global navigation satellite system) receivers. 

A large number of papers report some variations of ionospheric parameters before many large EQs, such as the F2-layer critical frequency (foF2) [36,85,86,87,88,89] and the sporadic E layer (Es) [90,91,92]. 

The study of foF2 alone is a very “inconvenient” ionospheric parameter for the role of EQ precursor, because, besides the geomagnetic activity effects, there would be many other reasons for non-EQ related foF2 variations. Therefore, in order to achieve a more robust result, a multi-parameter analysis is preferable and some works have analyzed more ionospheric parameters at the same time. For instance, in the periods of time preceding all crustal EQs in Central Italy with magnitudes M > 5.0 and the epicenter depth < 50 km, [88] considered the ionospheric sporadic E layer (Es) together with the blanketing frequency of Es layer (fbEs) and foF2, by analyzing data from the ionospheric observatory inside the preparation zone. According to these authors, the found deviations of ionospheric parameters from the background level can be related to the magnitude and the epicenter distance of the corresponding EQ. Very recently, the same procedure has been systematically applied for the first time to Greek earthquakes in the frame of the SAFE project [36]. Table 2 shows the confusion matrix of the statistics from which we can estimate the overall accuracy, A = 69%, hit rate of success, H = 50% and the false alarm rate, F = 26% (for their definition, please see, e.g., [93]). These values are encouraging because confirm the robustness of the technique and statistically prove the validity of the method, quantifying the higher significance of the found results with respect to casual events.

There is significant literature related to the analysis of the ionospheric effects before and during an EQ revealed by GPS/GNSS ground-based measurements, in terms of TEC fluctuations and scintillation anomalies that have been claimed to be detected some days before the EQs. Just to mention the more recent works, [94] analyzed 5 years of GNSS-based ionospheric TEC data by producing maps over an area surrounding the epicenter of the 2009 L’Aquila EQ. In the night of 16 March 2009, an interesting ionospheric anomaly was found, anticipating the main shock by 3 weeks, which could be connected with it. [95] reported on the analysis of the TEC from eight GPS stations of the EUREF network by using discrete Fourier to investigate the TEC variations over the Mediterranean region before and during the 12 October 2013 Crete, Greece EQ. Over an area of several hundred kilometers from the EQ epicenter, all stations used in this study observed an increase of 2-6 TECU from 10 October to 15 October 2013, likely related to the EQ. [96] applied a complex algorithm, the Firefly Algorithm (FA), as a robust predictor to detect the TEC seismo-ionospheric anomalies around the time of the some powerful EQs (27 February 2010 M8.8 Chile, 11 August 2012 M6.4 Varzeghan and 16 April 2013 M7.7 Saravan). Significant anomalies were observed 3–8 days before the EQs.

A recent paper by [97] presented the application of the LAIC model to compute the TEC variations and compare the simulation results with TEC observations for the Tohoku-Oki EQ (Japan, 11 March 2011, Mw 9.0). In the simulations, these authors assumed that the stress-associated current starts ~40 min before the EQ, and then linearly increases reaching its maximum magnitude at the time of the EQ main-shock. Comparisons with experimental values suggest that a dynamo current density of ~25 nA m^−2^ is required to produce the observed variation of ~3 TECU.

However, it is worth noting that the relationship between ionospheric anomalies and electromagnetic signals generated by the EQ preparation is still controversial and highly debated, as demonstrated by the high number of papers reporting a re-analysis of data and comments aiming to refute evidence of this correlation. For example, [98] commented on the findings of [99]. After a re-analysis of the data, used by Heki (2011) to demonstrate the existence of a TEC anomaly 40 min before the 2011 Tohoku-Oki EQ and other M > 8 EQs, [100] concluded that this anomaly was due to an artefact introduced by the choice of the definition of the reference line adopted in analyzing TEC variations. However, more recently [101] came back again to the question with a deeper and more convincing analysis that the change of TEC was real and not an artefact because for the 2011 Tohoku-Oki EQ the TEC change was simultaneous all over the globe.

### 10.2. Pre-EQ Ionospheric Evidence from In-Situ Measurements

Although many works on the possible pre-EQ effects in the ionosphere were performed with the early advent of satellites, it was with the DEMETER (Detection of Electro-Magnetic Emissions Transmitted from EQ Regions; 2004–2010) and CHAMP (CHAllenging Minisatellite Payload; 2000–2010) missions that most of the striking results were obtained.

DEMETER was a French micro-satellite operated by CNES and specifically designed to the investigation of the Earth ionospheric disturbances due to seismic and volcanic activities. It operated for more than 6.5 years of the scientific mission (2004–2010). The results from the analyses of this satellite dataset seem to have statistically proved definitively the existence of the LAIC, however, it is still needed to understand the deterministic details. Using the complete DEMETER data set [102], careful statistical studies were performed on the influence of seismic activity on the intensity of low-frequency EM waves in the ionosphere. The seismic database used for these analyses was constituted by several thousands of magnitude M5 + EQs occurred the satellite lifetime. In particular, the normalized probabilistic intensity obtained from the night-time electric field data was below the “normal” level, shortly (0–4 h) before the shallow (depth < 40 km) M5+ EQs at 1–2 kHz. Clear perturbations were observed a few hours before the EQs, as another example of “imminent” forecast: they are real, although they are weak and so far only statistically revealed. No similar effects were observed during the diurnal hours and for deeper EQs. It is interesting also to note that the spatial scale *R* of the affected area is approximately 350 km confirming relatively well the size of the EQ preparation zone estimated using the [16] formula. The main statistical decrease is observed at about 1.7 kHz, corresponding approximately to the cut-off frequency of the first transverse magnetic (TM) mode of the Earth–ionosphere waveguide during the night-time. An increase of this cut-off frequency effect would therefore necessarily lead to the decrease of the power spectral density of electric field fluctuations observed by DEMETER in the appropriate frequency range, meaning a lower height of the ionosphere above the epicenter of the imminent EQ. As the EM waves propagating in the Earth-ionosphere wave-guide are mainly whistlers, this means that their propagation is disturbed above the epicenters of future EQs, instead of a change of their intensities. 

Refs. [103,104] took advantage of the simultaneous measurements of these two satellites: they analyzed the electron density and temperature, ion density composition and temperature data from DEMETER ISL (Langmuir probe), ICE (electric field instrument) and IAP (plasma analyzer Instrument), together with CHAMP PLP data (electron density and temperature) and IONEX maps of vTEC (vertical TEC) from IGS (International GNSS Service). They investigated the ionospheric fluctuations related to the EQs occurred in September 2004 near to the south coast of Honshu, Japan [103] and Wenchuan EQ (M7.9) of 12 May 2008 [104]. The main result was the detection of a gradual enhancement of the EIA (Equatorial Ionospheric Anomaly) intensity starting one month prior to the event, reaching its maximum eight days before, followed by a decreasing behavior, very likely due to an external electric field generated over the epicenter affecting the existing **E×B** drifts responsible of the EIA.

Ref. [105] confirmed and improved the previous results on the full lifetime of the DEMETER satellite. Their main result is that there is a significant positive or negative deviation of ion density around five days before the earthquake occurrence within 200 km of the future epicenter.

By analyzing the magnetic data from Swarm satellites of the European Space Agency, a recent paper [106] found some important patterns before the April 25, 2015, M7.8 Nepal EQ, that resemble the same obtained from the seismological analysis of the foreshocks. 

Other two large earthquakes have been investigated by the same approach as [106]. They are the M7.8 16 April 2016 Ecuador [107] and M8.2 8 September 2017 Mexico earthquakes [108], confirming a particular pattern in the cumulative number of the Y magnetic component swarm anomalous tracks. In these two works, the turning point anticipates the earthquake of about 9 and 100 days, respectively. A comparison of the daily level of the geomagnetic field, electron density and electron temperature in the Dobrovolsky area by detailed time series analyses was applied by finding other possible evidence for ionospheric EM effects induced by lithospheric activity.

### 10.3. Pre-EQ Atmospheric Evidence

The improvement and increase of satellite remote sensing missions go back to the early 1980s. Since then, evidence of many types of infrared (IR) physics parameters have been recognized as useful to identify possible pre-EQ anomalies. Among them, the most cited are the brightness temperature (BT), outgoing longwave radiation (OLR), surface latent heat flux (SLHF), skin surface temperature (SST), and the atmospheric temperature at different altitudes. Although the topic is still debated or even controversial, many scientists agree that those parameters could change during the preparation phase of EQs and so they are regularly recorded by satellite at regional and global scales. [96,109,110] carried out studies where found variations of temperature or aerosols. [111] found a clear BT anomaly (BT corresponds to the temperature of a black body that emits the same intensity as measured), in correspondence of Lushan M7 EQ (China). On the other hand, OLR is the emission of the terrestrial radiation from the top of the Earth’s atmosphere to space; it is controlled by the temperature of the Earth and the atmosphere above it, in particular, by the water vapor and the clouds. [112] reported anomalies in this parameter days before the seismic events. SLHF describes the heat released by phase changes and shows an evident dependence on meteorological parameters such as surface temperature, relative humidity, wind speed, etcetera. SST is the temperature of the Earth’s surface at radiative equilibrium (usually, the interface between soil and atmosphere, on lands; it is identical to Sea Surface Temperature over the seas), in contrast with the meteorological definition of surface temperature measured by air thermometers which take readings at approximately 1 m above ground level. We will study the SST for the epicentral areas of the L’Aquila and Emilia main-shocks.

The nature of the detected IR anomaly as a real temperature change, or perhaps just an emission in the IR frequency band, is a debated issue. In a recent paper, [113] showed a clear thermal IR (TIR) anomaly preceding the 2009 M6.2 L’Aquila (Italy) EQ. The authors proposed a mechanism of generation of electric currents in the lithospheric rocks when they are under stress and a consequent IR irradiation with no actual temperature change (e.g., [114]). However, some recent works identified SLHF [115] and surface temperature anomalies [116] occurring before large EQs, thus supporting the possibility for some actual change of temperature too. However, according to Freund (personal communication), this is only an apparent paradox because any stimulated IR emission from vibrationally very “hot” systems is not a “clean” process. Eventually, the system will “thermalize”, meaning literally that each newly formed peroxy bond on the surface of the Earth will become a “hot spot”, surrounded by a small halo where the neighboring atoms have actually increased their Joule temperature. Although the exact cause of such temperature rise is still unknown, it is possible to definitely exclude the radon as a possible direct heat source, based on the results of laboratory experiments conducted by [117]. Ref. [118] resort to another role of radon as a possible indirect source: it could drive particle ionization and aerosol aggregation, where the latent heat release can cause the found increase in the atmospheric temperature. 

Application of particularly sophisticated techniques is mandatory to identify the anomalous signal in the TIR data. For instance, [119,120,121,122] propose some robust satellite techniques that take into proper account the past behavior of the signal under investigation: the typical seasonal and yearly background is computed and statistically significant deviation from it may represent the thermal anomaly. [123] focused the attention on the air-quality data as possible indicators of an impending EQ: these authors found a staggering increase in ambient SO_2_ concentrations by more than one order of magnitude across Taiwan several hours prior to two (M6.8 and M7.2) significant EQs in the island.

Although still controversial, an interesting emerging study concerns the EQ clouds [124], suggesting that their formation is due to some local weather conditions caused by energy and particle exchanges between crust and atmosphere. This process is believed to be able to locally modify the global electric circuit during the EQ preparation phase (e.g., [125]); or to create the conditions for electrical discharges in an atmosphere that may be the source of very high frequency (VHF) radio-emissions, sometime detected prior to large EQs [126]. Recently, the claim of unusual cloud formation prior strong EQs by [124] was strongly questioned by [127] with a counter-analysis based on examination of 4 years of satellite images and correlation analyses between linear-cloud formations and EQ occurrence.

### 10.4. Physical Models

**Figure 7:**
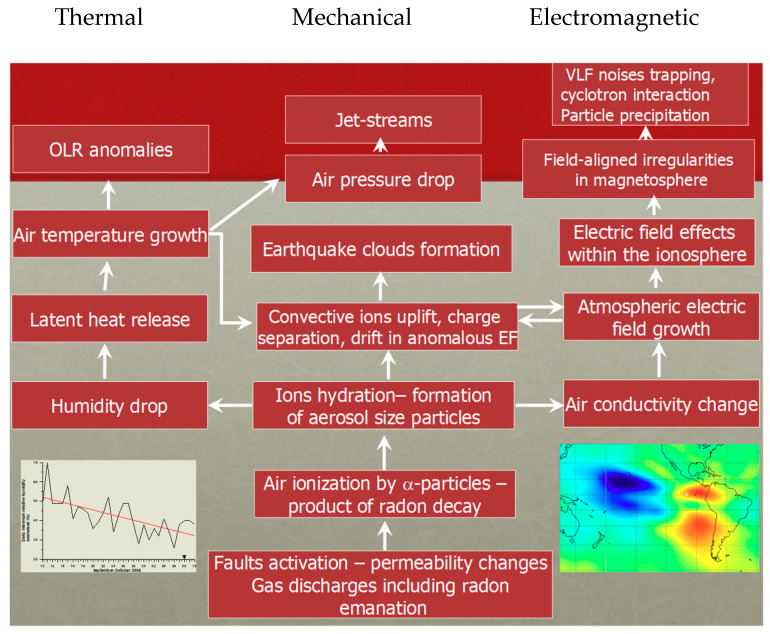
Pulinets-Ouzonouv LAIC (Lithosphere-Atmosphere-Ionosphere_Coupling) model (adapted from [118,128]).

A plausible physical omni-comprehensive model justifying the great variety of evidence given before is the real difficult conundrum for the scientists in this field. There are many theories that attempt to describe the physical processes manifesting anomalous behavior in some parameters before the occurrence of an EQ and try to explain what could cause these precursors. Several reviews of these processes can be found in [78,114,128] and the references therein (Figure 7). 

There exist many proposed mechanisms of generations to explain the LAIC, which can generally be classified as those based on mechanical (atmospheric waves generated by earth motions) and electrical (electric fields in Earth’s crust) sources: among the former, we can count the various kinds of atmospheric waves as internal or acoustic-gravity waves (IGW and AGW, respectively), planetary waves and tides. In particular, the hypothesis of acoustic-gravity waves generation before EQs was proposed by many authors (e.g., [78]; and more recently, [129]). 

The mechanisms that describe the anomalous electric field generation are more complex and intriguing. A theory that could explain many observations is based on the emission of radioactive gas or metallic ions before an EQ, which might change the distribution of electric potential above the surface of the Earth and then up to the ionosphere (e.g., [130]).

**Figure 8:**
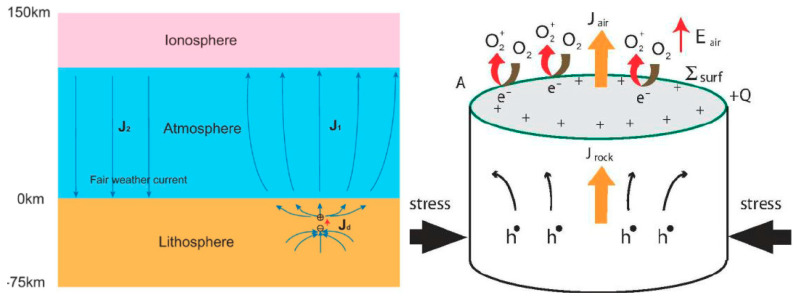
Freund model (adapted from [136]).

Whatever its source is, penetration of the electric field into the ionosphere could induce anomalies in the ionospheric plasma density and/or conductivity, which are observed above seismic zones (see e.g., [86,131]). In contrast with this view, [125] proposed that radon emitted before an EQ would increase the conductivity of air at ground level and that the ensuing increase of current in the fair weather global circuit would descend the ionosphere. This mechanism is also supported by [118]. However, [132] estimated that even if radon is coming out the ground in seismic areas, its contribution to the air conductivity is of minor importance relative to the air ionization rate, which can be expected from charge carriers from the rocks, the so-called positive-holes (or p-holes) (Figure 8). 

They showed experimentally that these mobile electric charge carriers flow out of the stressed rocks (see [132], and references therein) and, at the Earth’s surface, they cause extra ionization of the air molecules. However, the original experiments that detected these p-holes have been recently contrasted [133] (but see also [134]). 

Refs. [135,136] showed that ionospheric density variations could be induced by changes of the current in the global electric circuit between the bottom of the ionosphere and the Earth’s surface where electric charges associated with stressed rocks can appear. The interaction of the anomalous electric current with the geomagnetic field can even amplify the effect in the higher atmosphere [136]. 

**Figure 9:**
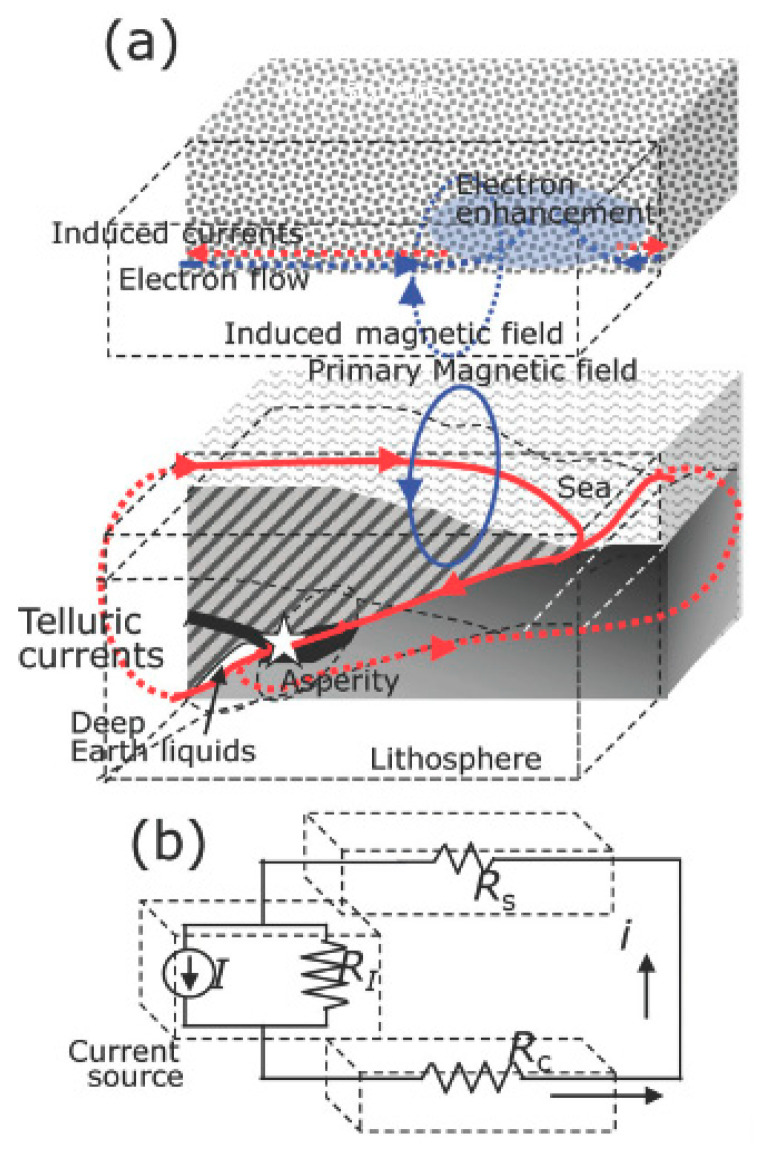
Enomoto model (adapted from [137]).

Ref. [137] introduced a fault model that takes account of the couple interaction between EQ nucleation and deep Earth gases and proposed a physical model of magnetic induction coupling with ionosphere before large offshore EQs (Figure 9). 

## 11. Examples of Thermal Coupling before L’Aquila and Emilia EQs

An important feature in the LAIC model should be the coupling between the lithosphere and the low atmosphere (i.e., the troposphere) in terms of thermal coupling. While the thermal coupling in case of volcanic eruptions is quite clear and convincing (e.g., [138]), that for earthquakes is more controversial. As was mentioned in the previous section, no general consensus exists about the fact that the thermal anomaly is just an infrared effect (e.g., [114]) or a real change of temperature (e.g., [116]). We do not want to express here a clear position in this debate. Rather, as didactical examples, we will show some SST studies for the same cases we analyzed for the entropy, i.e., the 2009 L’Aquila and the 2012 Emilia sequences of EQs.

In each case study, we will consider the SST in the epicentral region about two months around the EQ occurrence, and then we will compare the temperatures with those measured in the same day, at the same time (06:00UT) in the time interval 1979-2008 (2011) for L’Aquila (Emilia) EQ. An anomaly of the physical quantity of concern is defined as a value that exceeds the mean (or median) by two times the standard deviation and persists for at least two days (see also [35]). 

**Figure 10:**
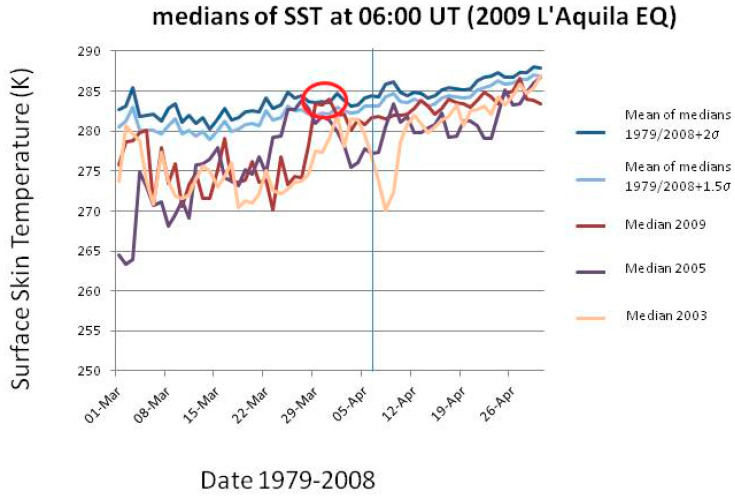
Median behavior of 2009 from 1 March to 30 April, compared with all 1979–2008 medians, and particular comparison with 2003 and 2005 medians. All values have been estimated at the epicenter. The red oval indicates when the thermal anomaly in 2009 is larger than or equal to 2 standard deviations, σ (as computed from the previous 1979–2008 years) and persists for at least two days. The vertical line is the EQ occurrence.

**Figure 11:**
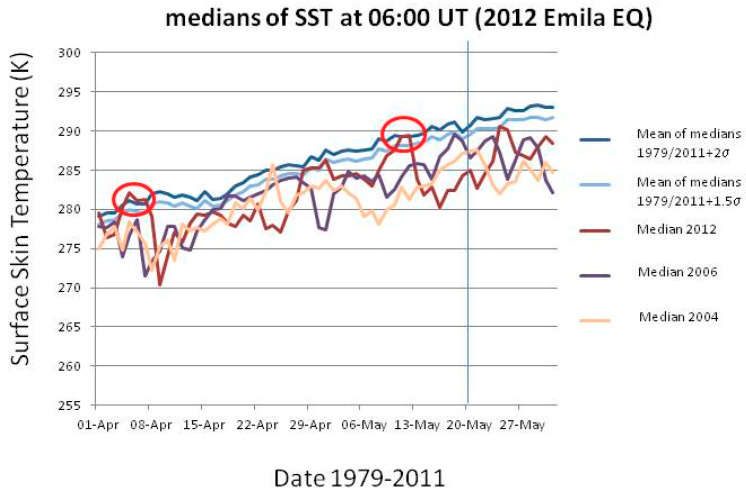
Median behavior of 2012 from 1 April to 31 May, compared with all 1979–2011 medians, and particular comparison with 2004 and 2006 medians. All values have been estimated at the epicenter. The red ovals indicate when the thermal anomaly in 2012 is larger than or equal to 2 standard deviations, σ (as computed from the previous 1979–2012 years) and persists for at least two days. The vertical line is the EQ occurrence.

Figure 10 and Figure 11 show the results for the two analyses. In detail, Figure 10 (Figure 11) shows for L’Aquila (Emilia) EQ the median behavior of 2009 (2012), from 1 March (April) to 30 April (31 May), compared with all 1979–2008 (2011) medians, and particular comparison with 2003 (2004) and 2005 (2006) medians. For each day, the use of the median was preferred because it was thought to be a more robust indicator. The latter years have been used for comparison because no significant seismicity occurred in those years in the two considered regions. All values have been estimated at the EQ epicenter. The red oval indicates when the thermal anomaly in 2009 (2012) is larger than or equal to 2 standard deviations, σ (as computed for each day from the previous 1979–2008 (2011) years) and persists for at least two days. In both analyses, a clear anomaly is found around a week before the EQ occurrence (vertical line in both figures). In the case of Emilia EQ, another persisting anomaly is also found around 1 month and a half before the main-shock.

These results confirm some previous studies on the possible thermal coupling in the two EQ cases (e.g., [113,116]). Central Italy showed an analogous thermal anomaly around 40 days before the recent 24 August 2016 M6 Amatrice EQ: [35] applied the CAPRI algorithm (CAPRI stands for “Climatological Analysis for seismic PRecursor Identification”) that removes the long-term trend over the whole day by day dataset. This procedure is used mainly to remove a possible “global warming” effect, avoiding to classify as abnormal a more recent year just because of global warming. These authors integrated the analysis of the skin temperature (skt) also with total column water vapor and total column of ozone and made a confusion matrix analysis for the last twenty years. As an example, Table 3 shows the confusion matrix of the validity of the skt as precursor applied to Central Italy earthquakes from 1994 to 2016. The following results are obtained: overall accuracy = 74%, hit rate of success = 40%, false alarms = 17%, (for more details please see [35]). These values confirm the validity of this thermal parameter as a potential pre-earthquake indication, at least for the area of concern, i.e., Central Italy. 

## 12. Mutual Information and Transfer Information: A Possible Future Direction

Geosystemics focuses on the inter-relations among the components composing the terrestrial complex system. For this reason, every statistical (or physical) quantity that measures these inter-relations is useful. However, given that the system under study is not usually linear, instead of linear quantities such as correlation coefficient or cross-correlation function between two variables belonging to linear processes, we have to resort to statistical quantities, which are more appropriate for nonlinear processes, as typical in a complex system.

Given two variables or time series X and Y, which characterize two processes of the phenomenon under study, we define the mutual information I(X, Y) extending definition (1) to two variables, i.e.,

(13) $$I(X;Y)=\sum_{y\in Y}\sum_{x\in X}^{}p_{}(x,y)\cdot \log\left(\frac{p(x,y)}{p_{1}(x)p_{2}(y)}\right)$$

where *p*_1_(*x*) and *p*_2_(*y*) are the corresponding probabilities and p(*x*,*y*) is the joint probability.

However, this formulation does not provide hints about the direction of information transfer between process X and process Y, i.e., from a part of a system to another. For this purpose, it is possible to introduce a useful definition that quantifies the information flow in terms of the Kullback and Leibler entropy [139], which can be defined for a single process X as:

(14) $$K_{x}=\sum_{x}^{}p(x)\cdot \log[p(x)/q(x)].$$

The above quantity is the entropy related to the process X when a different probability *q*(*x*) is used instead of the true *p*(*x*). We can also consider two different processes or variables and adapt the Equation (14) using conditional probabilities and taking into account a proper delay in one (see [31]). This gives rise to the so-called transfer information (changing its sign it becomes the transfer entropy) which provides knowledge not only about the information exchange but also about the direction of the information flow.

Here, we do not describe more details but we just want to emphasize the importance of quantifying direction of information flow amongst different parts or processes of the system under study. Often it is more important to know where the flow of information is going, instead of just estimating the information that is exchanged by the whole process between internal components or external ones [140].

Applying this concept to two different time series can be useful to say if one is the master quantity (that represents the causal process) and the other the affected one. [141] gave a very recent example of an application of this concept to geomagnetic field and climate.

In all cases, where we would like to compare/correlate a seismic sequence with possible atmospheric or ionospheric series of precursors, the calculation of the transfer information would provide a robust answer.

## 13. Conclusions

This paper has introduced the concepts of geosystemics [1,2,21] and then has shown its applications to some case studies. The spirit of geosystemics is to use some universal tools to look at some macroscopic quantities, such as the entropy, the Benioff strain, or the temperature to consequently deduce macroscopic properties of the physical system under study. An important frame is that of dynamical systems approaching a critical point when the macroscopic properties of the system change dramatically. This could be the case of a sequence of EQs that culminates with a main-shock. Therefore, we have shown some results obtained with the study of two recent Italian seismic sequences, the 2009 L’Aquila and the 2012 Emilia sequences. 

It is obvious that being the study of EQs a very complex problem, the more characterizing parameters are analyzed, the more robust the result will be. [67], for the case of the 2009 L’Aquila seismic sequence, gave a recent and extensive example of this approach.

A further question is how we can use the Big Data in geosciences and, in particular, to analyze precursory patterns of big earthquakes. Of course, the analysis of a greater number of data and the check of multiple models is perceptible that allows us to find some type of pattern before an earthquake, that could be likely valid only for regions very localized. An extensive statistical big data analysis would be important to confirm or confute the individual case results (although no definitive conclusions can be arisen because high correlation does not always mean causation; however can be of great help in proposing a physical framework of the chain of processes that could occur before a large earthquake). [35] gave an example of this approach, where the validity of local climatological variations as possible seismic precursors in Central Italy was statistically established.

Finally, we hope that this investigation can contribute to the worldwide scientific debate and efforts in understanding the earthquake preparation phase in order to arm the scientific community and stakeholders, against the natural disasters. 

## Figures and Tables

**Table 1:** Main data related to the two Italian seismic sequences under study: (from left to right) the label, the main-shock source parameters, the number of data points (foreshocks) used in the fitting stage; the maximum distance from the main-shock epicenter defining the selection area and the minimum threshold magnitude of the selected events there considered. We provide also a rough estimation of the predicted magnitude (within brackets) of the impending main-shock (see text). N and R in the Fault style column stand for Normal, and Reverse focal mechanism, respectively. R_max_ and M_min_ are the largest area and minimum magnitude, respectively, considered in the analyses of R-AMR, while for the entropy analyses we considered always the completeness magnitude (M1.4 and M2 for L’Aquila and Emilia Earthquakes).

	Sequence ID	L’Aquila	Emilia
**Main-shock Parameters**	Coordinate *(lat lon, in degree)*	42.34N 13.38E	44.89N 11.23E
	Depth (*km*)	8.3	6.3
	Date	6 Apr 2009	20 May 2012
	$t_{f}$ (in *days* from 1 May 2005) (predicted)	1436.06 (1437.4)	2576.09 (2577.7)
	Fault style	N	R
	*Magnitude (predicted)* *	5.9 (5.3 ± 0.5)	5.9 (5.7 ± 0.5)
	**# data (foreshocks)**	17	38
	**R**_**max**_ ** (km)**	300	300
	**M**_**min**_ (*)	4.0	4.0

* normally deduced from Equation (2a) or (2b).

**Table 2:** Confusion matrix for pre-earthquake anomaly detection obtained from ionospheric anomalies analysis in Greece from 2003 to 2015 (adapted from [36]).

Ionospheric Anomaly	Seismicity	
	Yes	No
Yes	5	9
No	5	26

**Table 3:** Confusion matrix for pre-earthquake anomaly detection obtained from skt time series analysis from 1994 to 2016 in Central Italy (adapted from [35]).

Skin Temperature Anomaly	Seismicity	
	Yes	No
Yes	2	3
No	3	15
